# On Staying Grounded and Avoiding Quixotic Dead Ends

**DOI:** 10.3758/s13423-016-1028-3

**Published:** 2016-04-25

**Authors:** Lawrence W. Barsalou

**Affiliations:** Institute of Neuroscience and Psychology, University of Glasgow, 58 Hillhead Street, Glasgow, G12 8QB UK

**Keywords:** Concepts and categories, Grounded cognition, Abstraction, Context

## Abstract

The 15 articles in this special issue on The Representation of Concepts illustrate the rich variety of theoretical positions and supporting research that characterize the area. Although much agreement exists among contributors, much disagreement exists as well, especially about the roles of grounding and abstraction in conceptual processing. I first review theoretical approaches raised in these articles that I believe are Quixotic dead ends, namely, approaches that are principled and inspired but likely to fail. In the process, I review various theories of amodal symbols, their distortions of grounded theories, and fallacies in the evidence used to support them. Incorporating further contributions across articles, I then sketch a theoretical approach that I believe is likely to be successful, which includes grounding, abstraction, flexibility, explaining classic conceptual phenomena, and making contact with real-world situations. This account further proposes that (1) a key element of grounding is neural reuse, (2) abstraction takes the forms of multimodal compression, distilled abstraction, and distributed linguistic representation (but not amodal symbols), and (3) flexible context-dependent representations are a hallmark of conceptual processing.

Although large literatures in cognitive psychology and cognitive science address the cognitive mechanisms underlying conceptual representation, this special issue of *Psychonomic Bulletin & Review* focuses heavily on the underlying neural mechanisms and whether these mechanisms are consistent with classic amodal theories or grounded cognition. Because these issues motivate much recent research, they receive the most attention in the special issue. As we will also see, however, traditional issues in cognitive psychology and cognitive science receive discussion as well and are important to integrate with recent neural issues in future research.

## Quixotic Dead Ends in the Study of Concepts

The study of concepts offers a significant set of challenges. First, conceptual processing proceeds largely unconsciously, such that it is difficult to observe concepts and their effects. Indeed, perhaps the dominant view on the relation between concepts and consciousness is that concepts operate completely unconsciously (for a review, see Kemmerer, 2015b). Second, as a consequence of operating unconsciously, concepts are difficult to discern in content, structure, and operation. By and large, researchers must use indirect methods to study them. Third, concepts operate across an unusually broad spectrum of intelligent activities, ranging from perception, to language and thought, to social and affective processes. How researchers view concepts in each of these areas can vary widely. Approaches to concepts in language, for example, often differ considerably from approaches in nonverbal domains, such as object and scene recognition. Fourth, as a result of the preceding challenges, concepts are associated with an unusually wide variety of theoretical proposals and positions. Indeed, it is difficult to think of a domain characterized by so many different views and so much disagreement (Barsalou, 2003b, 2012; Margolis & Laurence, 1999; McRae & Jones, 2013; Murphy, 2002).

Because of these challenges, there is the potential to head off in the wrong direction. At least many researchers in the area believe this, often accusing each other of being completely misguided in their approach. In this spirit, I begin with candidates for what I will refer to as *Quixotic dead ends*. In my opinion, pursuing these approaches is unlikely to yield success in understanding concepts, specifically, and cognition, more generally. Because these approaches often are principled, inspired, idealistic, and optimistic, while being impractical and unrealistic, I refer to them as Quixotic.

I do appreciate that what one researcher perceives as a Quixotic dead end is another’s ultimate solution. Thus, I recognize that I may be wrong about my predictions to follow. After presenting my predictions for approaches unlikely to succeed in this section, I turn to approaches that I believe are more likely to succeed in the next.

## Quixotic Dead Ends in Grounded Cognition

I begin with two approaches associated with grounded cognition that are unlikely to succeed as accounts of concepts (and of cognition more generally). I refer to these approaches and related ones later as “grounded,” not “embodied,” because “grounded” better captures the central focus of the general perspective by including other forms of grounding beside embodiment, such as multimodal simulation, physical situations, and social situations (Barsalou 2008a, Barsalou 2010; Kiefer & Barsalou, 2013).

### Simple sensory-motor accounts.

One possibility is that conceptual knowledge is completely constructed from sensory-motor representations, being reducible to them. A common theme across articles in the special issue, however, is that simple sensory-motor accounts won’t succeed. Not a single author in this issue proposes or defends this perspective (even though it often is attributed to grounded cognition researchers, as we will see). All authors who address the issue concur that sensory-motor mechanisms are insufficient for explaining concepts and conceptual processing (Binder, 2016; Dove, 2016; Jamrozik, McQuire, Cardillo, & Chatterjee, 2016; Leshinskaya & Caramazza, 2016; Reilly, Peelle, Garcia, & Crutch, 2016; Zwaan, 2016; also see Mahon, 2015).^1^ As these authors argue compellingly, extensive abstraction occurs throughout the human conceptual system, and sensory-motor mechanisms are unlikely to explain much of it (although sensory-motor mechanisms in the conceptual metaphor approach attempt to explain some of it; see Dove, 2016; Jamrozik et al., 2016). If there are actually any Don Quixotes wandering this neighborhood, they must have their noses close to the ground.

### Anti-representation accounts.

Another possibility—sometimes associated with the grounded perspective because of its emphasis on coupling the body with the environment during perception and action—is that cognition can be explained without the construct of representation. From this perspective, conceptual representations are theoretical fictions that are unnecessary for explaining cognition. One approach from this perspective assumes that cognition can be reduced to sensory-motor contingencies (Engel, Maye, Kurthen, & König, 2013; O’Regan & Noë, 2001). Another focuses on laws governing perception, action, and the body (Chemero & Turvey, 2011; Gibson, 1979). Although proponents sometimes seem convinced that these approaches will explain cognition, not everyone else is equally convinced, believing that representation is essential for successful accounts. For one reason, representational states appear necessary for explaining how learning can flexibly modulate the relation between stimuli and responses (Barsalou, in press-a; Chomsky, 1959; Lachman, Lachman, & Butterfield, 1979). For another, the ability to represent non-present states is central to what is unique and powerful about human cognition (Donald, 1993).

### Common caricatures and distortions of grounded approaches.

As described later, I believe that other approaches to grounded cognition hold promise for providing successful accounts of conceptual processing. My optimism about these approaches may indeed be Quixotic, but I nevertheless continue to view them as having potential. Interestingly, perhaps, these approaches are often caricatured and distorted by critics, turning them into, what I would agree, are Quixotic pursuits. I address several such caricatures and distortions presented in the special issue next.

Machery (2016) refers to grounded cognition with the endearing moniker of “neo-empiricism” (also see Machery, 2007). I agree that purely empiricist accounts, incorporating no biological constraints, are unlikely to be successful. Indeed, some grounded cognition researchers have explicitly expressed a commitment to nativist factors (Barsalou, 1999, 2008a; Simmons & Barsalou, 2003). Many classic nativists, such as Kant and Reid, centrally included sensory-motor processes, such as imagery, in their accounts (Barsalou, 1999, 2008a, 2010). Nevertheless, grounded cognition researchers continue to be called neo-empiricists, even though none of us to my knowledge explicitly rejects important native contributions. Strong biological constraints certainly exist on the cognitive system, including on conceptual processing. Consistent with Caramazza and Shelton (1998), for example, Simmons and Barsalou (2003) argued that feature and association areas in the brain have been shaped by evolution to anticipate important categories, such as foods, tools, and agents.

Leshinskaya and Caramazza (2016) state with considerable amazement that “extreme versions” of the “embodiment program,” such as Barsalou (2008a) and Pulvermüller and Fadiga (2010), continue to “claim that concepts are entirely reducible to modality-specific sensory or motor representations.” Leshinskaya and Caramazza further state that Barsalou, Simmons, Barbey, and Wilson (2003) endorse the reduction of concepts to sensory-motor representations, citing the following quote as evidence,

“Theories generally assume that knowledge resides in a modular semantic system separate from episodic memory and modality-specific systems for perception, action and emotion. These theories further assume that conceptual representations are amodal—unlike representations in modality-specific systems—and operate according to different principles. Increasingly, researchers propose that conceptual representations are grounded in the modalities.” (p. 84)

First, it is difficult to see how the above quote makes any kind of reductionist claim; it simply claims that knowledge is grounded. Second, neither Barsalou (2008a) nor Pulvermüller and Fadiga (2010) claimed that concepts can be reduced to sensory-motor representations. To the contrary, Barsalou (2008a, p. 620) stated that,

“Grounded theories are often viewed as only using sensory-motor representations of the external world to represent knowledge. As a result, it is argued that grounded theories cannot represent abstract concepts not grounded externally. Importantly, however, embodiment researchers since the classic empiricists have argued that internal states such as meta-cognition and affect constitute sources of knowledge no less important than external experience.”

Throughout later sections, Barsalou (2008a) continues to describe how both language and internal states contribute to the representation of concepts above and beyond the sensory-motor modalities. Analogously, Pulvermüller and Fadiga (2010) never claimed that cognition can be reduced to sensory-motor representations but instead focused on how action supports perception and how perception-action relations *contribute* to language.

Second, both Barsalou and Pulvermüller clearly claim throughout their published articles that other mechanisms besides sensory-motor representations are essential for explaining concepts. Pulvermüller (2013), for example, argued that disembodied mechanisms in the brain’s hub regions contribute to semantic meaning (Pulvermüller, 2012; Pulvermüller & Garagnani, 2014). Similarly, Barsalou (1999, pp. 585, 600-603) went to considerable lengths in proposing that internal states play central roles in conceptual processing, especially for abstract concepts (also see Barsalou & Wiemer-Hastings, 2005). In a different vein, Barsalou, Santos, Simmons, and Wilson (2008) reviewed a program of research showing that distributed linguistic representations play central roles in representing and processing concepts. Given the large numbers of publications from both groups going beyond sensory-motor mechanisms, it is difficult to understand how Leshinskaya and Caramazza could have missed them.

In concluding their article, Leshinskaya and Caramazza make a rather ironic proposal about how the field should be “Moving forward.” Specifically, they propose that future research should focus on nonsensory attributes of knowledge, such as intention, belief, and function. To provide background for their recent research, Leshinskaya and Caramazza cite Wilson-Mendenhall, Simmons, Martin, and Barsalou (2013) as having previously illustrated this approach (i.e., by establishing non-sensory-motor attributes associated with the concepts of *convince* and *arithmetic*). Notably, Wilson-Mendenhall et al. (p. 921) cited four previous articles going back to Barsalou (1999) as background for the hypothesis that “the lexical representation for “convince” is associated with much nonlinguistic semantic content that supports meaningful understanding of the concept, including the intentions, beliefs, internal states, affect, and actions of self and others that unfold in a spatio-temporal context.” Using neuroimaging, Wilson-Mendehall et al. established the neural areas that underlie this abstract non-sensory-motor content. Given that Leshinskaya and Caramazza cited this work, their earlier claim that Barsalou (2008a) and Barsalou et al. (2003) reduce concepts to “modality-specific sensory-motor representations” is puzzling. It’s been clear for some time that our group views abstract representations as essential.

Goldinger et al. (2016) offer a host of common misconceptions about grounded approaches, warping them into Quixotic dead ends. I address only a few of the more salient misconceptions. First, Goldinger et al. focus on embodiment in embodied cognition. Although embodiment is an important theme of grounded cognition, it is certainly not the only theme or arguably the most important one. Most, if not all, grounded cognition researchers would agree that embodiment is far from sufficient for explaining cognition and believe that it may be irrelevant during much cognitive processing (Barsalou, 2008a, 2010). The crux of the grounded approach is understanding how the modalities, the physical environment, the social environment, *and* the body *contribute* to cognition, playing central roles in the diverse forms it takes (Barsalou, Breazeal, & Smith, 2007).

Second, Goldinger et al. state that “modeling is currently impossible in EC [embodied cognition].” Without a doubt, any approach that can’t be modeled is a non-starter. Review articles, such as Barsalou (2008, pp. 634-635), however, often have cited computational models of grounded cognition, which continue to accumulate (Adams et al. 2014; Blouw, Solodkin, Thagard, & Eliasmith, 2015; Caligiore, Borghi, Parisi, & Baldassarre, 2010; Eliasmith, 2013; Pulvermüller & Garagnani, 2014; Schrodt, Layher, Neumann, & Butz, 2015; Thagard & Stewart, 2011). Given the existing literature, how could anyone responsibly claim that computational models aren’t possible?

Goldinger et al. further propose that grounded cognition is not sufficiently specified to produce concrete models. Notably, all other approaches to cognitive theory must address this issue as well, including the classic information processing perspective, neural nets, Bayesian modeling, and so forth. For each of these general approaches, it is necessary to make assumptions that constrain the space of models, thereby making the construction of specific models possible. Information processing, for example, is so general that a huge space of models exists, many of which are implausible in humans, such as many models in machine learning, other areas of computer science, and mathematics. Only when assumptions relevant to human information processing are made, does it become possible to construct meaningful models about human cognition. Grounded cognition is no exception, with the models developed so far reflecting various theoretical commitments that made them possible.

Third, Goldinger et al. suggest that grounded cognition aims to replace standard theories of cognition but is unable to do so. In my experience, re-explaining all basic cognitive phenomena is not something to which grounded cognition researchers aspire *at all*. Again, most, if not all, grounded cognition researchers would agree that grounded cognition, on its own, is far from capable of explaining everything that we know about cognition. To the contrary, grounded cognition complements traditional approaches. Rather than aiming to replace traditional approaches, grounded cognition develops their relations with the modalities, the body, and the environment (Barsalou, 2008a, p. 635-636). Similarly, an integration of grounded approaches with classic symbolic and statistical approaches is likely and necessary (Barsalou, 1999, p. 652; Barsalou, 2010, p. 721).

Finally, Goldinger et al. present a long list of basic cognitive phenomena, such as word frequency, short-term memory scanning, and serial recall, arguing that the grounded approach hasn’t informed our understanding of them. Goldinger et al.’s surprisingly lengthy (and elementary) descriptions of these phenomena give the “copious theoretical writing” of grounded cognition a run for its money. No grounded cognition researcher would argue that grounded cognition should or could re-explain all cognitive phenomena. Again, grounded cognition aims to complement and incorporate theoretical constructs and empirical findings in the field, such as those on Goldinger et al.’s arbitrary list.

In summary, I completely agree that the approaches to grounded cognition covered in this section are dead ends, including approaches that have no biological constraints, approaches that assume concepts are reducible to sensory-motor systems, approaches that focus completely on the body, and approaches that aim to replace existing cognitive theories rather than complement them.

## Quixotic Dead Ends Associated with Amodal Symbols

I turn next to another class of theories that I believe are equally unlikely to succeed, namely, theories proposing that amodal symbols constitute the central form of conceptual representation. I will assume that the amodal symbols discussed in this section have two properties: (1) they are arbitrarily related to their corresponding categories in the world and experience; and (2) they can stand alone without grounding to perform the basic computations underlying conceptual processing. In a later section, I entertain other types of abstract representations having different properties that strike me as more plausible.

### Machery’s (2016) *offloading hypothesis*.

According to this account, concepts consist of amodal symbols, and conceptual processing results from operating on them. When it is necessary to relate these results to the world, the amodal system offloads them to sensory-motor systems, which then perform various tasks. Perhaps this is a way to build a robot (although not the smarter and more interesting robots that roboticists have been building for the past 30 years). Nevertheless, I’m betting that this is not how the human brain works. Based on arguments to follow here and in later sections, the offloading hypothesis strikes me as the epitome of a Quixotic dead end.

### Leshinskaya and Caramazza’s (2016) *interaction hypothesis*.

Similar to the offloading hypothesis, Leshinskaya and Caramazza make a clear distinction between concepts and sensory-motor processing, with some brain areas supporting concepts, and others supporting sensory-motor processing. Like Machery’s offloading hypothesis, conceptual processing interacts with sensory-motor processing when necessary, but doesn’t depend on it in any crucial ways. In other words, the representations in these conceptual areas (presumably amodal symbols) are again sufficient for stand-alone conceptual processing. When processing color features in concepts (e.g., yellow for banana), a brain region that represents conceptual information about color becomes active, but no use of visual color areas is necessary. Conversely, when color is perceived in visual areas, it causes the retrieval of amodal representations of color in the related conceptual area, which can represent color without use of the visual areas. Because this approach assumes that conceptual processing areas only interact with sensory-motor areas when necessary, it is essentially no different than Machery’s offloading hypothesis. The clear implication of this proposal is that different brain areas underlie concepts and sensory-motor processing, contrary to the basic proposal of grounded cognition that systems used in sensory-motor processing are also used to some extent during conceptual processing (as described later in the section on neural reuse).

Interestingly, the interaction hypothesis often (but not necessarily) assumes that conceptual areas lie near related sensory-motor areas. To explain why conceptual processing often activates regions within sensory-motor processing streams, the interaction hypothesis proposes that conceptual processing areas utilize amodal symbols that reside within these streams. When processing color conceptually activates areas in the color perception pathway (Hsu, Frankland, & Thompson-Schill, 2012; Simmons et al., 2007), the interaction hypothesis proposes that these activated areas contain amodal symbols. In this way, it becomes possible to argue that activations for conceptual processing in sensory-motor streams don’t actually offer support for grounded views. Instead, these activated areas implement conceptual processing using amodal symbols that interact with nearby sensory-motor areas when necessary.^2^

### Mahon's (2015) *default explanation*.

Like Machery (2016) and Leshinskaya and Caramazza (2016), Mahon (2015) assumes that there is a clear distinction between conceptual areas and sensory-motor areas, with conceptual areas utilizing amodal symbols. Indeed, he concludes that,

“The core issue at stake in the discussion about whether concepts are embodied has been resolved: concepts are represented in an amodal format” (p. 424).

Similar to Leshinskaya and Caramazza (2016), Mahon adopts the interaction hypothesis, referring to it as *grounding by interaction* (also see Mahon & Caramazza, 2008). Again, conceptual knowledge and sensory-motor processes reside in different brain areas and interact when necessary, with interactions between them playing central roles in conceptual processing. Nevertheless, Mahon (2015), like Machery (2016), believes that conceptual processing can function in a stand-alone manner, stating that,

“The human mind must have something like a clutch: something that allows thinking to proceed unencumbered by our representations of our body and the world” (p. 421).

Going a step further in this line of argument, Mahon (2015) presents an account of temporal dynamics called *the default explanation*, whereby conceptual processing always occurs first in conceptual areas and then may later spread optionally to sensory-motor areas as a function of context (pp. 422, 426-427). Mahon (2015) further claims that this is the default view of conceptual processing, given that it has been widely established and accepted (even though important alternative views have existed for decades; see the discussion of core-first, core-last, and no-core theories in Lebois et al. 2015; also see Casasanto & Lupyan, 2015; Connell & Lynott, 2014; Yee & Thompson-Schill, 2016).

Finally, Mahon (2015) concludes that researchers must first reject the default explanation before attempting to show that alternative grounded theories have merits—the “burden of proof” is on these other theories (p. 424). Implicit in this argument is the assumption that no further evidence is required to accept the default explanation. The only empirical question is whether new evidence can be found that rejects it and/or that supports alternative accounts. As we will see, this implicit assumption has given amodal theories an unfair advantage when it comes to evaluating evidence and arguments for various theories.

In the process of making these claims, Mahon forces a wide variety of views onto his Procrustean bed. According to him, for example, the theories of Barsalou (1999) and Simmons and Barsalou (2003) contain amodal symbols in convergence zones. As described later, these theories actually propose that patterns of conjunctive neurons in convergence zones integrate modality-specific content in a topographical manner, thereby implementing multimodal compression, not amodal symbols. Similarly, Mahon argues that Martin (2007, 2009) endorses the interaction hypothesis. As Martin’s (2016) contribution to the special issue clearly indicates, he believes instead that the brain areas active during conceptual processing are essential parts of modality-specific processing streams and do not perform stand-alone conceptual processing:

“my position is, *and has always been*, that the regions where we store information about specific object-associated properties are located within (i.e., *overlap with*) perceptual and action systems, specifically excluding primary sensory–motor regions.” [italics added]

By forcing these diverse views into the default explanation, Mahon is contributing to the collection of caricatures and distortions that the amodal position has produced about grounded cognition.

In recent years, Martin (personal communication) has been asking researchers who use the term “amodal” what they mean by it. Overwhelmingly, he finds that they mean *multimodal*, not amodal. Sloppy use of “amodal” has resulted in this confusing state-of-affairs. To move forward, it is essential to distinguish carefully between different forms of abstraction, with amodal symbols constituting one particular form. The later section on abstraction reviews three other forms of abstraction, besides amodal symbols, that contributors to the special issue suggest: multimodal compression, distilled abstraction, and distributed linguistic representation. Lumping these four significantly different forms of abstraction together as “amodal” is unlikely to yield progress in understanding how the brain implements conceptual processing.

In general, two basic assumptions of amodal approaches make me skeptical that they offer correct and useful accounts of human conceptual processing: (1) the arbitrary redescription of modality-specific content into amodal symbols, and (2) using these symbols to perform conceptual processing in a stand-alone manner (at least on some, if not many, occasions). I continue to doubt that the brain contains amodal conceptual representations that are arbitrarily related to modality-specific representations and to their corresponding referents in the world (Barsalou, 1999). Like Damasio (1989), I don’t see evidence of any brain regions that implement these kinds of representations, and I don’t see any compelling rationale for why they would. Furthermore, I don’t see a compelling rationale, much less any evidence, for the proposal that amodal symbols can operate in a stand-alone manner to perform conceptual processing. For these reasons, I view the amodal approaches reviewed in this section as likely to be Quixotic dead ends. Later sections flesh out my skepticism further.^3^

### Black holes in conceptual space.

An intriguing aspect of the amodal position is that it never provides concrete descriptions of what amodal concepts are or how they are supposed to work. None of the articles offered by proponents in this collection even point to specific proposals, much less begin to develop them. Machery (2016), for example, simply defines amodal concepts as having a format that “differs from the format or formats of perceptual and motor representations,” a remarkably uninformative definition. Thus, I have become fond of referring to amodal concepts as *black holes in conceptual space*. Amodal concepts appear to have considerable gravitational pull for some researchers, yet are so mysterious, dark, and dense as not to be visible and describable. Additionally, the gravitational fields surrounding these black holes appear to generate considerable distortion in perception, as we have seen.

Seriously, how can we begin to understand and test amodal theories if it is not clear what they are? Perhaps these researchers simply assume that Fodor's (1975) Language of Thought is such an obvious solution that they do not need to mention it (i.e., the view that cognition results from symbolic operations on structured amodal representations independently of the modalities and physical situations)? It is this problem, more than any other, that makes me believe that the amodal approach is heading up a Quixotic dead end. At least Reilly et al. (2016) are honest about the problem,

“Foremost, the neurobiological mechanisms by which hubs perform propositional transformations remain essentially a black box.…We must currently take it on faith that the language of thought involves a form of mental calculus that operates over abstract symbols: we have only the most rudimentary understanding of how the brain extracts and manipulates symbols.”

As we have seen, the offloading hypothesis, the interaction hypothesis, and the default explanation all assume that the brain areas containing amodal symbols can support stand-alone conceptual processing. Given the importance of these brain areas in amodal theories, it is essential that clear coherent accounts of how they work are forthcoming, as well as empirical predictions that follow. The fact that such accounts do not exist constitutes a serious cause for reservation about this class of theories.

### If prediction *X* from grounded cognition is false, then the amodal view must be true.

As we saw earlier, part of being associated with the default explanation is having a privileged status when it comes to evidence. All other things being equal, the amodal view is most likely true by default, especially when evidence for grounded cognition falls through. No direct evidence for the amodal view is necessary. Consider a couple of examples. If an action verb fails to activate motor areas in an experiment, grounded cognition must be false, and the amodal view must be true. Analogously, if a motion verb fails to activate motion areas, again grounded cognition must be false and the amodal view true. For articulation of this position, see Machery (2016).

Clearly, action and motion verbs do activate areas associated with action and motion, respectively, at least on some occasions (for many examples of supporting findings, see Dove, 2016, Jamrozik et al., 2016, and Kemmerer 2015a).^4^ Importantly, however, action and motion verbs do not activate motor and motion areas automatically on all occasions (Tomasino & Rumiati, 2013). This well-established finding has led some researchers to draw the default conclusion that if grounded representations are not always active, then amodal concepts must constitute the conceptual cores of concepts that *do* become automatically active across contexts (Dove, 2009; Machery, 2007; Mahon & Caramazza, 2008).

An emerging theme across multiple literatures, however, is that concepts do not have conceptual cores, and that even the most salient features of concepts are not activated automatically (Gawronski & Cesario, 2013; Kiefer, Adams, & Zovko, 2012; Lebois et al., 2015; Santiago, Román, & Ouellet, 2011; also see Moors & De Houwer, 2006). Not only is this true of grounded features, such as action and visual motion, it appears true of all features, including color features in the Stroop task, positional features in Simon tasks, numerical features in SNARC tasks, and so forth. Such results have increasingly led researchers toward the view that conceptual processing is highly flexible, as many articles in the special issue illustrate (Binder, 2016; Dove, 2016; Kemmerer, 2015a; Yee & Thompson-Schill, 2016; Zwaan, 2016; also see Mahon, 2015). Rather than being active in every context, even the most central features of a concept vary in how active they are across contexts. Most importantly, it doesn’t follow from the varying activity levels of grounded features that grounded theories are false (Lebois et al., 2015). Dynamic theories that include grounding naturally explain the finding that the activation of grounded features varies across contexts. Even more importantly, it doesn’t follow by default that amodal theories are correct given these results, because no direct evidence for amodal symbols has been offered.

### If double dissociations in lesion patients occur, then the amodal view must be true.

Another instance of the default logic concerns double dissociations in lesion patients. First, find patients who have intact sensory-motor processing but have conceptual deficits related to these sensory motor processes (e.g., intact motor processing with deficits in action concepts). Second, find patients who have sensory-motor deficits but intact conceptual processing (deficits in motor processing but intact action concepts). Should these two kinds of patients exist, then grounded views must be false, and amodal views must be true. In the special issue, this logic is endorsed for action concepts (Machery, 2016; Leshinskaya & Caramazza, 2016; Reilly, Peelle, Garcia, & Crutch, 2016; also see Mahon, 2015) and for food concepts (Rumiati & Foroni, 2016).

There are two problems with this logic. First, it adopts the dead end perspective on grounded cognition that conceptual processing is simply equated with sensory-motor processing. Given this perspective, take away a sensory-motor process, and any concept that depends on it must no longer exist. Second, this logic again assumes that if a prediction for the grounded view fails, the amodal view must be true. It’s not necessary to actually show that amodal symbols represent concepts, because this is the default explanation.

Several contributors to the special issue point out that once plausible grounded views are adopted, it becomes easy to explain double-dissociations (Kemmerer, 2015a; Martin, 2016; Yee & Thompson-Schill, 2016): When lesion patients have a deficit on one sensory-motor modality, they use another partially redundant modality to perform a related conceptual task. When processing the conceptual meaning of motor verbs, for example, patients with motor deficits might comprehend motor verbs using visual motion information. Similarly, when processing the meanings of food concepts, patients with odor deficits might comprehend food concepts using visual and taste information. Another possible explanation follows from adopting an architecture that contains hierarchically-organized convergence zones grounded in primary feature areas, with some convergence zones being modality-specific and others being category-specific (Simmons & Barsalou, 2003). Depending on where a lesion falls, numerous combinations of deficits and spared abilities can occur, including the double dissociations just described. Still another explanation follows from the distributed linguistic representations described later, with linguistic representations flexibly supporting conceptual processing in a compensatory manner when a related modality is damaged.

Although these accounts have been in the literature for years, and even though multiple contributors to the special issue point to such accounts, the double dissociation argument lives on. Not only do double dissociations fail to reject plausible grounded views, the default explanation simply doesn’t follow without clear and compelling empirical evidence for amodal symbols.

Having said all this, grounded views generally predict that significant damage to a modality should have consequences for conceptual processing. If conceptual processing relies on a modality—as argued later for neural reuse—then it should change in some way after the modality is damaged. To assess such consequences, however, it is first necessary to rule out uninteresting and uncontrolled confounding factors. If, for example, a double dissociation doesn’t control for the compensatory use of other modalities, association areas, and/or distributed linguistic representations, it is impossible to assess the consequences of a damaged modality. Furthermore, assessments of the damaged modality should be sufficiently sensitive and well-designed to establish these consequences. Without designing double dissociation research around these basic principles, it is impossible to assess the theoretical implications of damage to a modality rigorously.

### The fallacy of using conjunction analysis with multimodal cues to establish amodal symbols in the brain.

Another questionable attempt to support amodal views comes from a strategy pursued in recent neuroimaging experiments (Devereux, Clarke, Marouchos, & Tyler, 2013; Fairhall & Caramazza, 2013; Van Doren, Dupont, De Grauwe, Peeters, & Vandenberghe, 2010). The basic idea is as follows. First, identify different cues for a concept that differ in modality-specific ways (e.g., the spoken word for a concept such as “dog” vs. a picture of a specific dog instance). Second, present each cue to participants and establish the parts of the brain that carry information about the associated concept from that cue. Third, use conjunction analysis to establish brain areas that carry information about the concept for *both* cues. Fourth, conclude that these shared areas contain amodal symbols. Thus, the crux of this approach is that any brain area carrying information about a concept when activated by cues from different modalities must represent the concept amodally (yet another case of the default explanation). Machery (2016) argues that this kind of evidence demonstrates the clear existence of amodal symbols. Reilly et al. (2016) offer a specific example, arguing that brain areas active for both visual and auditory cues implement amodal symbols.

The obvious problem with this logic is its failure to recognize that different cues could all activate the same modality-specific information, not just shared amodal symbols. The word “dog” and a picture of a dog, for example, could both activate visual motion areas that represent motion properties associated with dogs in a grounded manner. If so, then the conjunction analysis has identified shared modality-specific information, not the concept’s amodal core. Because researchers performing these experiments typically do not entertain this possibility, they don’t attempt to establish whether shared areas in conjunction analyses process modality-specific or amodal representations. Thus, again, we do not have compelling evidence for amodal symbols in the brain, further leading to the conclusion that this class of theories is heading down a Quixotic dead end.

## Promising Approaches to the Study of Concepts

My pessimism about some approaches represented in the special issue is more than tempered by my optimism about others. The large majority of contributions point to future directions that are likely to be productive, leading to greater understanding of concepts, specifically, and of cognition, in general (including contributions of which I’ve been critical so far). I turn next to themes raised in these contributions that, in my opinion, are likely to be useful elements of future research, including grounding, abstraction, flexibility, temporal dynamics, the ability to explain classic conceptual phenomena, and making contact with real-world situations.

## Grounding

Many of the contributions in the special issue acknowledge the importance of grounding conceptual processes and further acknowledge the considerable evidence for grounding that has accumulated (Binder, 2016; Dove, 2016; Hauk, 2016; Jamrozik et al., 2016; Kemmerer, 2015a; Martin, 2016; Reilly et al., 2016; Yee & Thompson-Schill, 2016; Zwaan, 2016). Because some contributions raise issues about what is meant by grounding (Leshinskaya & Caramazza, 2016; Martin, 2016; Mahon, 2015), I begin with what I mean by it.

To a large extent, grounding concerns itself with the grounding problem raised initially by Searle (1980) and Harnad (1990), which asks how amodal symbols, specifically, and cognition, more generally, are linked to the modalities, body, and environment. In a review of research on grounding, Barsalou (2008a) argued that researchers have attempted to ground concepts and cognition by establishing their relations with modality-specific systems, the body, the physical environment, and the social environment (also see Barsalou, 2010; Barsalou et al., 2007; Kiefer & Barsalou, 2013). Thus, at a general level, grounding simply refers to programmatically studying cognition in new ways. Rather than studying cognitive mechanisms in isolation, establish their relations with the contexts in which they are embedded and on which they depend. At more specific levels, grounding refers to establishing specific accounts of how cognitive processes in the brain utilize the modalities, the body, and the environment. It does *not* mean reducing concepts and cognition to anything, including sensory-motor mechanisms. As described next, one important form of grounding is neural reuse, where cognition partially utilizes the modalities to implement its basic functions (but does not reduce to them).

### Neural reuse and simulation.

As we saw earlier, the offloading and interaction hypotheses assume that separate (and possibly adjacent) brain areas are used for conceptual and modality-specific processing, such that conceptual processing is not grounded—it is self-sufficient, not depending on any other systems (Leshinskaya & Caramazza, 2016; Machery, 2016; Mahon, 2015). According to this perspective, processing in conceptual areas doesn’t depend in any way on modality-specific processing but is simply linked to it.

An alternative view is that conceptual processing depends on modality-specific systems, utilizing the same—not different—systems that support perception, action, and internal states, what Anderson (2010) called *neural reuse.* As Martin (2016) describes for the domains of color and taste, conceptual processing in these domains partially utilizes the same systems used for visually perceiving color and for gustatorally perceiving taste (see Wang et al., 2013, for another thoughtful discussion of this issue). Obviously, the neural systems in conceptual processing and perception differ, given that the brain is implementing two different processes. Conceptual processing, for example, is more likely to draw on integrative and abstractive mechanisms in association areas (Binder, 2016; Simmons & Barsalou, 2003). As a consequence, conceptual processing does not reduce to modality-specific processing but utilizes many other systems as well. Nevertheless, depending on task conditions, conceptual processing, in part, often reuses systems that underlie perception, action, and internal states.

Neural reuse differs significantly from the idea that amodal symbols *describe* modality-specific information in stand-alone conceptual processing areas (e.g., amodal symbols that describe color and taste features; Leshinskaya & Caramazza, 2016; Mahon, 2015). Instead, neural reuse makes the stronger commitment that modality-specific information is represented conceptually by partially reusing the same brain areas that represent this information during perception and action. Thus, representing color and taste features conceptually requires reusing some of the same systems active during vision and eating.

Neural reuse offers a natural account of what is meant by *simulation* (Barsalou, 1999, 2003b, 2009): Reusing a modality-specific pathway during conceptual processing simulates the kind of processing that this pathway performs during perception, action, and/or internal states. Again, reuse may not be complete and may vary considerably across tasks and contexts. Nevertheless, the point remains that higher cognitive processes are grounded in more basic processes by virtue of reusing their neural resources. From this perspective, cognition cannot be performed by only using independent amodal symbols. Instead, it depends on modality-specific processes to some extent (along with other mechanisms such compressed multimodal representations, distilled abstractions, and distributed linguistic representations, as discussed later).

To the extent that neural reuse plays central roles in conceptual processing, much work must be performed to better understand it. Specifically, how are conceptual uses of modality-specific pathways related to uses of these pathways during perception and action? Are common areas used in similar manners, or in different manners? What computational functions do these areas perform, and how are they related to modality-specific and conceptual processing? What are the roles of different laminar layers for bottom-up and top-down input? In general, we need more sophisticated theoretical accounts of neural reuse that go beyond the simple proposal that neural reuse occurs. Analogously, we need to go beyond simple demonstration experiments and develop more systematic empirical investigations that test and inform these theories.

### Revisiting the interaction hypothesis.

When the first neuroimaging results on object concepts were reported, many researchers were surprised that they implicated modality-specific regions for vision and action (for reviews of such findings, see Martin, 2007, 2016; Martin & Chao, 2001). Many researchers also were surprised when behavioral research similarly implicated these regions in language comprehension (for reviews, see Zwaan, 2004, 2016; Zwaan & Madden, 2005). As this surprise illustrates, researchers expected to find representations of conceptual knowledge outside the brain areas that support modality-specific processing. It would not have been surprising, for example, if the neural activity underlying conceptual processing primarily resided in the higher-level association areas that Binder (2016) reviews, or in the anterior temporal lobes central to hub-and-spoke theories (Reilly et al., 2016). Furthermore, because one of the primary sources of brain expansion has been in association areas—not in the modalities—it might be expected that association areas would implement the relatively powerful conceptual abilities of humans, evolutionarily speaking (Buckner & Krienen, 2013).

For all of these reasons, arguing that brain regions with amodal symbols reside in what have traditionally viewed as modality-specific processing streams is unusual and counterintuitive. Yet, as we saw earlier, this is the claim of the interaction hypothesis (Leshinskaya & Caramazza, 2016; Mahon, 2015). According to this account, amodal regions in modality-specific processing streams can perform stand-alone conceptual processing, only interacting with adjacent modality-specific areas as needed.

In defending this claim, Leshinskaya and Caramazza (2016) and Mahon (2015) state that grounded researchers are not justified in making claims about the representational format used in regions of modality-specific streams active during conceptual processing. For example, Leshinskaya and Caramazza (2016) state,

“We do not see what neural organizing principles—either the nature of divisions or their location—can tell us about the nature of those representations.”

It follows that grounded researchers are not justified in claiming that activations for conceptual processing in these streams are modality-specific. Because the formats of the representations in these streams cannot be established, these representations could potentially be amodal instead. Notably, however, the focus of grounded researchers has typically been on the neural reuse of these regions for conceptual processing, *not* on their representational format. Barsalou (1999, p. 582), for example, dismissed the importance of format and stressed the centrality of neural reuse,

“Perceptual symbols are not like physical pictures; nor are they mental images or any other form of conscious subjective experience. As natural and traditional as it is to think of perceptual symbols in these ways, this is not the form they take here. Instead, they are records of the neural states that underlie perception. During perception, systems of neurons in sensory-motor regions of the brain capture information about perceived events in the environment and in the body.”

From this perspective, capturing records of neural states allows later reusing them for a wide variety of cognitive purposes, without making any commitment to their format (for discussion of reusing captured neural states, see Barsalou, 1999 pp. 603-608, 2008a, 2009).

Ironically, Leshinskaya and Caramazza (2016) and Mahon (2015) don’t follow their own admonitions about the inability to specify format, proposing that conceptual processing areas in modality-specific streams have an amodal format (or a non-sensory-motor format). Again, the default explanation has its privileges. Regardless, these proposed amodal regions continue to have the character of black holes in conceptual space. No accounts are provided of why these regions contain representations in an amodal format, much less hypothesis-driven empirical evidence for these claims. Instead, complex elaborate explanations based on questionable assumptions are used to justify them.

Martin (2016) similarly argues that we can’t determine the representational format of specific brain regions in modality-specific processing streams. Nevertheless, he states,

“Representations are grounded by virtue of their being situated within (i.e., partially overlapping with) the neural system that supports perceiving and interacting with our external and internal environments.”

Consistent with neural reuse, conceptual processing utilizes modality-specific resources, such that conceptual processing overlaps with modality-specific processing. It is not the format that matters but the fact that conceptual processing utilizes modality-specific resources—whatever their formats happen to be. Conceptual processing does not operate in a stand-alone manner but instead relies on regions of modality-specific pathways. Martin (2016), together with many others in the special issue issue and elsewhere, review considerable evidence consistent with the reuse perspective (for some recent examples, see Schwiedrzik, Bernstein, & Melloni, 2016; Waldhauser, Braun, & Hanslmayr, 2016).

One final point about format: Regions of a modality-specific pathway, such as the ventral stream, have probably evolved to perform specific types of computations on relevant information. As a consequence, the representations and processes in these regions are likely to reflect such constraints. As a further consequence, representations and processes are likely to differ across modalities (e.g., vision vs. audition vs. action vs. affect vs. taste). If so, then representations and processes within a particular modality-specific processing stream are modality-specific, not amodal. The fact that cytoarchitectonics vary within and across modalities is consistent with this proposal (Amunts & Zilles, 2015), as is the fact that different conceptual information can be represented in different cytoarchitectonic areas (Grill-Spector & Weiner, 2014). Thus, if the neural reuse hypothesis is correct, it follows that when a conceptual process utilizes the resources of a modality-specific processing stream, the resultant conceptual representations have a modality-specific character, not an amodal one.

Bringing cytoarchitectonics into the discussion may make it possible to address the format issue in a much more sophisticated way than simply being concerned with whether a brain area contains amodal or iconic (sensory-motor) representations. The key issues are likely to be, first, establishing the neural computations that the cytoarchitectonics of an area make possible, and second, establishing the cognitive functions these computations realize. I suspect that once we understand these computations, they will have little to do with the issue of amodal vs. iconic format. Instead, understanding these computations is likely to play central roles in understanding how conceptual processing reuses modality-specific pathways.

### Grounding and cognitive processes.

Goldinger et al. (2016) refer to the “poverty of embodied cognition.” As Mahon's (2015) Figure 1 illustrates, articles on embodied cognition have been growing exponentially since 1980 (this count apparently doesn’t include articles on grounded and situated cognition). Clearly, some researchers view the grounded perspective as having value, and perhaps for reasons described so far: It’s important to understand how cognition is related to the modalities, to the body, and to the environment, especially if cognition depends on them and doesn’t operate independently. Many researchers believe that the chances of understanding cognition will increase if we incorporate grounding.

Alternatively, concern exists that cognitive psychology is not evolving in important new ways, but has become stuck in an approach that hasn’t changed much in decades. Traditionally, cognitive psychologists focus on cognitive processes per se, ignoring how they’re grounded (e.g., the classic processes of information processing, such as attention, working memory, lexical access, etc.). Studying these processes typically occurs in highly idealized laboratory paradigms designed for the sake of establishing the properties of a particular process, not because these paradigms reflect important behaviors in the world that we need to understand better. When modeling findings from these paradigms, the resultant theories typically have little relevance for explaining how the cognitive processes of interest manifest themselves in real-world behavior. Thus, we end up with theories of laboratory paradigms whose relations to the world are tenuous. As a further consequence, we accumulate a long list of processes, like those reviewed in Goldinger et al.’s article, with little understanding of how they relate to anything, including the modalities, the body, and the environment. One might worry that this poverty of riches is leading the field down a Quixotic dead end.

Grounding offers a natural direction for developing accounts of basic cognitive processes. Again, grounded cognition primarily aims to complement existing accounts, not to replace them. Not only does grounded cognition benefit from incorporating basic cognitive processes, it offers new opportunities for studying them, and for understanding how they operate when embedded in the modalities, body, and environment. As Barsalou (in press-a) suggests, cognition can be defined as the mediating processes between stimuli and responses that make adaptive action in goal-directed situations possible. As Barsalou (in press-c) further suggests, cognition is organized around a situation processing architecture that underlies a wide variety of cognitive, social, affective, and appetitive behaviors, with neural reuse and simulation playing central roles. Examining basic cognitive processes from grounded perspectives like these offers new opportunities for understanding, studying, and explaining their underlying mechanisms and their roles in human intelligence.

Perhaps new insights into basic cognitive processes will develop, despite Goldinger et al.’s skepticism. Several of the topics Goldinger et al. cover—priming, face processing, and sentence processing—have received considerable attention in grounded cognition, with researchers believing that something new has been learned (Lebois et al., 2015; Niedenthal, Mermillod, Maringer, & Hess, 2010; Santiago et al., 2011; Zwan, 2016).

## Abstraction

As researchers have become increasingly convinced that concepts are grounded, they have simultaneously become increasingly aware of how extensively abstraction is associated with conceptual processing. Indeed, abstraction appears to be a hallmark of human cognition and an important source of its computational power. Thus, a current challenge is explaining how grounding and abstraction emerge together.

It’s perhaps first worth noting that abstraction takes many forms. Barsalou (2003a), for example, listed six forms of abstraction often associated with conceptual processing: (1) categorical knowledge, (2) the ability to generalize, (3) summary representation, (4) schematic representation, (5) flexible representation, and (6) abstract concepts. Dove (2016) similarly addresses various forms of abstraction. Contributors to the special issue bring up all these forms at one point or another across articles (Binder, 2016; Dove, 2016; Jamrozik et al., 2016; Leshinskaya & Caramazza, 2016; Martin, 2016; Murphy, 2016; Reilly et al., 2016; Yee & Thompson-Schill, 2016; Zwaan, 2016; also see Mahon, 2015).

More significantly, these contributors suggest several mechanisms that could potentially implement abstraction, which differ in important ways from amodal symbols: multimodal compression, distilled abstraction, and distributed linguistic representation. Although Mahon (2015) reduces these different mechanisms into a single construct of amodal symbols, this move does not offer a useful way forward. Rather than seeing these mechanisms as the same, it is important to understand the differences between them and to establish their consequences for explaining results, motivating future research, and for informing our understandings of the brain and cognition. Certainly, amodal symbols offer one way of implementing abstraction. Nevertheless, multimodal compression, distilled abstraction, and distributed linguistic representation offer important alternatives that appear easier to define theoretically and that enjoy stronger support empirically. They also all appear highly compatible with grounded approaches, as we will see.

### Multimodal compression in convergence zones.

One approach to abstracting over the multimodal instances of a category is to compress their detailed content into a more general representation. In the special issue, Binder (2016) proposes that the brain contains a hierarchical organization of convergence zones, also frequently referred to as *association areas* and *hubs* (e.g., Buckner & Krienen, 2013; Sporns, 2010). Of primary interest for Binder’s account are cross-modal convergence zones whose conjunctive neurons integrate lower-order information across modalities (also see Damasio, 1989; Meyer & Damasio, 2009; Simmons & Barsalou, 2003). Within these convergence zones, patterns of conjunctive neurons establish *cross-modal conjunctive representations* (CCRs) that function as abstract representations. In the limit, Binder assumes that CCRs can become so abstract as to sometimes become amodal symbols. More typically, however, he assumes that they maintain information about the lower-order representations they integrate across modalities, thereby exhibiting data compression rather than arbitrary transduction (Barsalou, 1999). Rather than being arbitrarily related to multimodal simulations, as for amodal symbols, CCRs evolve out of multimodal simulations, continuing to carry information about them in a representational manner (perhaps this is the crux of what is meant by a *convergence* zone). In this spirit, Simmons and Barsalou (2003) proposed that conjunctive neurons are organized topographically within convergence zones, where topographical closeness can be defined by conceptual similarity as well as by modality-specific similarity. Binder further suggests that CCRs support the computations underlying categorical representations, thematic relations, propositions, and schemata.

Binder reviews much literature that bears on the localization of multimodal compressions, such as CCRs, in the brain. Specifically, this literature suggests that the associative processes underlying conceptual knowledge consistently engage brain areas outside modality-specific areas, including medial prefrontal cortex (dorsal and ventral), posterior cingulate cortex, angular gyrus/supramarginal gyrus, lateral middle temporal gyrus, inferior frontal gyrus, and ventromedial temporal cortex (also see Legrand & Ruby, 2009). In general, these areas tend to become more active for stimuli associated with many abstractions than for stimuli associated with fewer abstractions. Specifically, these areas tend to become more active for meaningful words than for pseudo-words (and for meaningful sentences and texts than for anomalous ones). The same areas are more active for familiar proper names and high-frequency words than for unfamiliar proper names and low-frequency words, and when highly related and associated concepts are processed together than when less related and associated concepts are processed together. Finally, processing concepts deeply activates these areas more than when the same concepts are processed shallowly.

Based on these findings, Binder concludes that these association areas process abstractions. As the abstractions associated with a stimulus increase, these areas become more active, because they represent and process the increasing numbers of abstractions. Additional findings from Fernandino et al. (in press) further implicate compressed multimodal representations in association areas, showing that association areas all over the brain track the modality-specific content of concepts. Rather than being arbitrarily related to modality-specific content in concepts, association areas represent it.

Compressed representations, such as CCRs, are essentially the same kind of representations as prototypes in cognitive theories of concepts (Murphy, 2016). According to prototype theory, statistically likely features are extracted from category exemplars and conjoined in a prototype that represents the category conceptually (Hampton, 2006; McRae, Cree, Seidenberg, & McNorgan, 2005; Rosch & Mervis, 1975; Smith & Medin, 1981). Notably, prototypes are not amodal symbols arbitrarily linked to examplars. Instead, the features of exemplars appear in the prototype that covers exemplars, following various possible forms of data compression, as for CCRs.

An alternative form of data compression that could underlie multimodal abstractions reduces exemplar information to a new set of dimensions (as in PCA, ICA, NMF, and so forth). Rather than using features from exemplars to represent a prototype, new dimensions are abstracted that capture correlated sets of features. As a result, different vectors across these new dimensions would represent concepts in Binder’s association areas. Again, these compressions aren’t amodal symbols, because they carry information about the information in exemplars. For examples of how this type of compression can be used to represent different kinds of concepts, see Reilly et al. (2016), Blouw et al. (2015), and Mitchell et al. (2008) . Many neural net models similarly use dimensional reduction to represent knowledge (Ganguli & Sompolinsky, 2012; Hinton, 2006).

As Murphy (2016) notes, exemplar theories offer an alternative to prototype theories, with exemplar theories assuming that individual exemplar memories represent a category instead of an aggregate prototype. Although Murphy has strong inclinations to view exemplar theories as a Quixotic dead end, the mechanisms that integrate exemplars into a category representation could potentially reside in the areas that Binder (2016) associates with abstraction, if these theories turned out to be accurate.

Murphy further suggests that exemplar *effects* offer a plausible alternative to exemplar *theories*, with salient exemplars sometimes controlling categorization (Allen & Brooks, 1991). A class of accounts that naturally explains both prototype and exemplar effects assumes that abstractions include statistical information about *correlations* of features, not just about *individual* features (Barsalou, 1990; Barsalou & Hale, 1993; McClelland & Rumelhart, 1985; also see Love, Medin, & Gureckis, 2004). Because exemplars can be viewed as patterns of correlated features, storing correlational information from them in abstractions implements exemplar effects (e.g., using hidden units; Gotts, 2016). Factorization approaches, such as PCA, ICA, and NMF, also are readily capable of capturing exemplar effects, representing salient exemplars with individual components. Again, such patterns constitute data compression that could plausibly be implemented in Binder’s (2016) association areas. As all these approaches indicate, it is possible to capture the exemplar effects that Murphy endorses, without further endorsing exemplar models that posit individual exemplar representations in memory.

### Distilled abstractions.

In their account of metaphor, Jamrozik et al. (2016) suggest another mechanism for abstraction that differs from multimodal compression. Rather than compressing multimodal information into a sparser representation, abstraction distills abstract features from more concrete ones, leaving behind a representation that contains only abstract features. Although Jamrozik et al. focus on abstract features transmitted into a concept via metaphorical comparison, in principle, any concept that contains both concrete and abstract features could have its concrete features filtered, leaving an abstract representation behind (cf. Barsalou, 1999, pp. 583-585).

What sorts of abstract features exist that could be distilled into abstractions? As discussed earlier, recent research has already established that the brain represents a variety of abstract features associated with the concepts of *convince, arithmetic, goal, belief*, and *function* (Leshinskaya & Caramazza, 2016; Wilson-Mendenhall et al., 2013). The abstract features of *reward* and *magnitude* offer further examples of important abstract features, given that dedicated brain areas represent them to some extent, namely, the orbital frontal cortex and intraparietal sulcus, respectively (Rudebeck & Murray, 2014; Walsh, 2003; Wilson, Takahashi, Schoenbaum, & Niv, 2014). Potentially, many diverse abstract features structure experience extensively and thus play central roles in distilled abstraction.

Linguistic analyses of syntactic and semantic primitives offer another possible source for discovering abstract features. Talmy (1985), for example, offered numerous examples of abstract features that are lexicalized across languages, playing a wide variety of important roles in syntax and semantics, including features for cause, aspect, valence, together with more concrete features such as path, motion, and manner (also see Langacker, 1986, 2008, and many other cognitive linguists). Increasing research establishes the neural systems that implement these features (Kemmerer, 2006; Kemmerer, Castillo, Talavage, Patterson, & Wiley, 2008).

What is the status of abstract features in the brain? Because these features represent abstract information present in a situation, perhaps they are similar in status to sensory-motor features, such as color, motion, pitch, taste, touch, and movement that represent features in perception and action. As instances of these features are experienced, the corresponding feature areas become active to represent them. Alternatively, amodal symbols could represent abstract features, a common assumption that many researchers make. Nevertheless, it seems important and potentially useful to consider the possibility that abstract features have a similar status in the brain as sensory-motor features, coding aspects of experience directly, rather than being represented by amodal symbols that have been somehow transduced from experience (Barsalou, 1999; Barsalou & Wiemer-Hastings, 2005; Wilson-Mendenhall et al., 2013).

On subsequent occasions, when abstract features of entities and events are situations are represented conceptually, the areas associated with processing abstract features are reactivated, simulating what those situations were like abstractly. Similar to sensory-motor features, neural reuse supports the conceptual processing of abstract features. Many of these features are likely to have strong biological origins (e.g., *magnitude, reward, mental state*).

A key issue is whether abstract features reside in the association areas that Binder (2016) reviews or elsewhere. Preliminary evidence suggests that they can reside in both. Consider findings from Wilson-Mendenhall et al. (2013). For the abstract concept, *arithmetic,* they found activations in the intraparietal sulcus that overlapped with activations for a numerical localizer task. Because this brain area does not fall within Binder’s association areas, and because it is often associated with the representation of magnitude, it follows that some abstract features are processed by dedicated feature areas. Again, it seems important to enumerate these kinds of features, to establish where they reside in the brain, and how they contribute to abstractions.

Wilson-Mendenhall et al., however, observed a different finding for the activations associated with the abstract concept, *convince.* In general, activations for these areas fell largely into Binder’s association areas (with some exceptions). Because these activations also overlapped with activations for a mental states localizer task, it is not clear whether the activations for *convince* reflect data compression versus distilled abstraction for features of mental states. Thus, another important issue for future research is to establish whether Binder’s association areas (and association areas more generally) perform data compression, represent abstract features, or both.

### Distributed linguistic representations.

In the special issue, Zwaan (2016) suggests that distributed linguistic representations can function as symbolic placeholders, which become fleshed out by multimodal simulations when greater information about a concept is needed. Although this account is similar to multimodal compression, it assumes that a completely different kind of representation—associated sets of words—function as a compressed account of conceptual content rather than conjunctive patterns in a convergence zone at the end of a processing stream. Many other researchers have made similar claims, arguing that distributed linguistic representations play central roles in conceptual processing (Andrews, Frank, & Vigliocco, 2014; Andrews, Vigliocco, & Vinson, 2009; Barsalou et al., 2008; Connell & Lynott, 2013; Glaser, 1992; Louwerse, 2011; Louwerse & Connell, 2011; Paivio, 1986); also see research on Latent Semantic Analysis and related approaches (Baroni & Lenci, 2010; Erk, 2012; Erk & Padó, 2008; Landauer & Dumais, 1997; Landauer, McNamara, Dennis, & Kintsch, 2013; Padó & Lapata, 2007). Because the words that constitute distributed linguistic representations typically take auditory, motor, and visual forms, they are modality-specific representations, not amodal symbols.

In general, this approach proposes that computations over distributed linguistic representations can be used to perform cognitive tasks effectively, although many researchers suggest that the effectiveness is less than when using multimodal simulations and other forms of conceptual knowledge (Barsalou et al., 2008; Connell & Lynott, 2013; Glaser, 1992; Louwerse & Connell, 2011; Zwaan, 2016). In other words, distributed linguistic representations offer a heuristic for performing a task quickly, when conditions permit, but may not be sufficient for sophisticated task performance. Again, they can be viewed as abstractions that carry information about more complex conceptual representations.

### Establishing the distributed networks that underlie concepts.

According to previous sections, a concept utilizes neural resources that represent features in modality-specific systems, together with various kinds of abstract features. Furthermore, these features feed into association areas that compress incoming information, perhaps at multiple levels, resulting in compressed abstractions. Abstractions of a concept can also result when abstract features are distilled from concrete ones, and when distributed linguistic representations are established for it.

If this account of concepts is correct, then establishing the distributed networks that implement neural reuse, data compression, distilled abstraction, and distributed linguistic representations presents a number of research challenges. One important challenge is to establish the association areas that play central roles in abstraction. Because a variety of conflicting proposals exist, assessing their relative merits is essential to producing a compelling account of these distributed networks.

Hub-and-spoke theories constitute one important class of theories. According to these theories, the most important association areas for concepts reside in the anterior temporal lobes (ATL) of both the left and right hemispheres (Reilly et al., 2016; also see Lambon Ralph, Sage, Jones, & Mayberry, 2010; Patterson, Nestor, & Rogers, 2007; Rogers & McClelland, 2004). From this perspective, the ATLs contain stand-alone amodal symbols that can perform conceptual processing on their own, but that project to sensory-motor areas when fleshing out amodal symbols is useful (similar to the interaction hypothesis). Although hub-and-spoke theories assume that the abstractions in the ATLs are amodal, the ATLs also could also potentially implement multimodal data compressions and/or distilled abstraction.

Perhaps a more serious problem for hub-and-spoke theories than its assumptions about amodal symbols is that many researchers do not view the ATL as a single homogeneous region for representing all concepts in general, but rather as a highly differentiated region where distinct concepts, such as those for social information and individuals, are represented separately from other concepts (Drane et al., 2008, 2009, 2013; Martin, Simmons, Beauchamp, & Gotts, 2014; Wong & Gallate, 2012). Furthermore, when Fernandino et al. (in press) used conjunction analysis to establish common association areas active across diverse concepts, the ATLs were not present. Binder (2016) suggests that other association areas in the temporal and parietal lobes are most likely to perform “hub” functions (also see Binder, Desai, Graves, & Conant, 2009). Martin (2016) similarly suggests that other temporal and parietal areas operate as hubs.

Once the association areas for conceptual processing are established, it then becomes important to establish whether they support data compression, distilled abstraction, distributed linguistic representation, or some other function (including amodal symbols). Once the operative representations in an associative area are established, another critical issue is determining whether these representations can operate in a stand-alone manner or can only operate together with modality-specific representations. When abstractions and sensory-motor simulations operate together, the nature of their interactions becomes important, along with how these interactions accomplish various conceptual functions (similar to issues associated with the interaction hypothesis).

Following Jamrozik et al. (2016), distilled abstractions can probably operate as stand-alone representations, given that they sometimes become active in metaphor without the support of activations in modality-specific areas (which can accompany them optionally). As Zwaan (2016) points out, however, abstract representations often become much easier to understand, once they have been embellished with modality-specific information (also see Akpinar & Berger, 2015; Schwanenflugel, 1991).

Distributed linguistic representations may also be capable of functioning as stand-alone representations, following many recent empirical demonstrations (Zwaan, 2016; also see Barsalou et al., 2008; Connell & Lynott, 2013; Louwerse & Jeuniaux, 2010). Again, they may only provide heuristic information about a concept, with more definitive representation requiring the addition of multimodal simulations.

Finally, it is not clear that multimodal compressions in association areas can function as stand-alone representations during conceptual processing, or whether they require support from modality-specific areas. One proposal is that compressed representations primarily serve to index, integrate, and control distributed representations across multimodal systems rather than to represent them in a stand-alone manner (Damasio, 1989; Simmons & Barsalou, 2003). Nevertheless, because multimodal compressions carry information about distributed multimodal representations, they could potentially be used in a stand-alone manner, at least for heuristic purposes.

### Temporal dynamics.

As Hauk (2016) notes, various distributed networks just considered make temporal predictions about neural activity and behavior. Hub-and-spoke theories, for example, predict that hub representations typically become active before spoke representations. Similarly, the default explanation proposes that conceptual areas become active before sensory-motor areas. Finally, research on distributed linguistic representations has shown that, at least under some conditions, distributed linguistic representations become active before conceptual representations, such as multimodal simulations.

Another possibility—consistent with research on flexibility discussed shortly—is that the networks underlying concepts can be accessed in diverse ways, such that no strict time course characterizes their operation. In this spirit, Zwaan (2016) illustrates how the processing of an abstract concept could differ when used cataphorically versus anaphorically (i.e., when an abstract concept is processed before or after a relevant situation in which it applies). When processed cataphorically, a sparse representation is accessed initially (perhaps a distributed linguistic representation), which is then fleshed out in modality-specific detail with the text that follows. Conversely, when processed anaphorically, relevant modality-specific detail is established before the abstract concept is presented, such that a rich conceptual representation is available immediately. To the extent that such flexibility exists, taking tasks and contexts into account is essential for making predictions about temporal dynamics.

Predictions about temporal dynamics offer an important tool for confirming and disconfirming hypotheses about the distributed networks that underlie concepts. Localization of these networks spatially is obviously important but limited in the information provided. Assessing temporal dynamics not only offers additional means of discriminating theories, it also forces researchers to articulate their theories more clearly. Developing detailed models of the types that Gotts (2016) explores is likely to support making sophisticated predictions about temporal dynamics.

### Abstract concepts.

Many contributors to the special issue note the importance of abstract concepts in assessing theories of concepts (Dove, 2016; Jamrozik et al., 2016; Leshinskaya & Carmazza, 2016; Martin, 2016; Reilly et al., 2016; Zwan, 2016; also see Mahon, 2015). These contributors often note that abstract concepts are ubiquitous in human cognition, perhaps playing more important roles than concrete concepts. Even more often, abstract concepts are presented as a specific challenge for grounded theories, even though they actually constitute a major challenge for all theories. To my knowledge, the amodal approach has offered even less insight into abstract concepts at this point than grounded approaches, given the current literature. Again, the default assumption often is that if the grounded approach cannot explain something, then the answer must be amodal. So goes the common story about abstract concepts. Regardless, the amount that we understand about abstract concepts from *any* perspective is shockingly modest. Simply claiming that abstract concepts are represented by amodal symbols does not explain anything about their content, structure, and function.

Abstract concepts seem like a particularly hard nut to crack. Our lack of understanding doesn’t reflect a lack of effort but more likely reflects what a hard problem this is. If we want to understand human concepts—and indeed human cognition—it is essential that we establish some traction in understanding abstract concepts. I suspect that all the abstraction mechanisms described earlier play important roles. Because abstract concepts often appear to integrate information across situations, they may utilize multimodal compression. Because they often include abstract features, they may utilize distilled abstraction. Because they appear highly dependent on language (Binder, Westbury, McKiernan, Possing, & Medler, 2005), they may utilize distributed linguistic representations. Consistent with the multifaceted character of abstract concepts, both Dove (2016) and Jamrozik et al. (2016) propose that multiple mechanisms are likely to underlie them.

Moving beyond our modest understanding of abstract concepts may require studying a few of them in detail, rather than studying them in the aggregate, especially given their diversity (Wilson-Mendenhall et al., 2013). Because abstract concepts appear dependent on background situations (Akpinar & Berger, 2015; Schwanenflugel, 1991; Zwaan, 2016), examining their operation in specific situations may provide leverage—and perhaps be necessary—for making significant progress.

## Flexibility

Another major theme across contributions to the special issue is that concepts operate flexibly, with their representations and effects depending on tasks and contexts (Binder, 2016; Dove, 2016; Kemmerer, 2015a; Yee & Thompson-Schill, 2016; Zwaan, 2016; also see Mahon, 2015). In their review of context effects on concepts, Yee and Thompson-Schill illustrate that the processing of a concept varies as a function of long-term context, recent context, immediate context, ongoing context, and individual abilities. They further argue that (1) concepts cannot be separated from the contexts in which they occur (consistent with context availability theory; Schwanenflugel, 1991), (2) concepts are not rigidly fixed in content, and (3) concepts do not have conceptual cores. Yee and Thompson-Schill further suggest that recurrent neural networks offer a natural way of explaining how all the different levels of context shape the knowledge underlying a concept and its changing content over a processing episode.

Kemmerer (2015a) first reviews evidence that motor and motion areas frequently become active to represent the meanings of action and motion verbs. Then, focusing on action verbs, he shows that activations in motor areas do not always occur when processing them. Analogous to Yee and Thompson-Schill, he reviews a wide variety of factors that modulate motor activations during meaning activation. Motor activations only represent verbs, for example, under conditions when they are relevant and available for doing so. Otherwise, verb meanings are represented flexibly using other mechanisms along the lines of those just discussed for abstraction. After briefly reviewing evidence that color information is not always active in the Stroop paradigm, Kemmerer states,

“Surely, the lack of a consistent interference effect in the Stroop paradigm does not imply that the color features of color words are not genuine components of the meanings. By the same token, the discovery that the motor features of action verbs (and tool nouns) are not always accessed in the same way does not imply that those features are not genuine components of the meanings.”

Based on a review of automaticity in the semantic processing literature, Lebois et al. (2015) similarly argue that concepts don’t have conceptual cores that are activated immediately across all contexts. Instead, even the most central features of a concept can vary widely in activation. They further propose that the flexible activation of information in a concept reflects Bayesian sampling: On a given occasion, the probability that a given feature of a concept is active reflects (1) the overall frequency with which the feature has been processed in previous contexts (its prior) and (2) its relevance in the current context (its likelihood). Although frequent features have a high chance of becoming active, context can override them, conferring an advantage on contextually relevant features. As context becomes increasingly salient and specified, contextually relevant features may become increasingly dominant, similar to well-known context effects in the Visual World Paradigm (Tanenhaus, Spivey-Knowlton, Eberhard, & Sedivy, 1995; for a recent review see, Huettig, Rommers, & Meyer, 2011) Based on a wide variety of such findings, Hauk (2016), Yee and Thompson-Schill (2016), and Lebois et al. (2015) suggest that understanding the dynamic activation of features for a concept in context over time should be a central topic of future research (for a recent example of such work, see van Dam, Brazil, Bekkering, & Rueschemeyer, 2014).

Finally, Gotts (2016) provides elegant and insightful reviews of repetition priming in behavior, together with the corresponding repetition suppression in neural activity, exploring the implications for incremental learning. Although the models that he reviews and develops primarily address the processing of individual stimuli, the underlying mechanisms have clear relevance for the representation of conceptual knowledge, as he notes. In addition, these mechanisms provide a natural way to understand and model the kinds of conceptual flexibility that Kemmerer (2015a), Yee and Thompson-Schill (2016), and Lebois et al. (2015) review. Our understanding of conceptual flexibility will be enhanced significantly to the extent that we can explain the phenomena reviewed in these articles with the kinds of models that Gotts develops. Implementing such models using the network architectures motivated earlier in the sections on grounding and abstraction offers one direction for model development. Because these architectures implement multiple forms of representation across association areas and modalities, a given concept can be flexibly activated in many different ways.

## Explaining Classic Conceptual Phenomena

In the special issue, Binder (2016) and Murphy (2016) remind us that theories of concepts must explain classic conceptual phenomena (Barsalou, 2003b, 2012; Margolis & Laurence, 1999; McRae & Jones, 2013; Murphy, 2002). As Binder reviews, concepts play important roles in taxonomies, thematic relations, propositions, and schemata. Murphy also addresses the importance of taxonomies, along with basic level categories, exemplar effects, and category-based induction. In particular, Murphy emphasizes the importance of causal reasoning and background knowledge in conceptual processing. Typicality and all its effects would be another basic phenomenon that I’d add to this list.

I’d further add basic symbolic operations, such as type-token binding, predication, propositions, productivity, and concept composition (Fodor, 1975; Fodor & Pylyshyn, 1988; Pylyshyn, 1984). Although it might seem that symbolic operations are only relevant to amodal theories, and can only be explained by them, I continue to believe that they fundamentally structure human cognition, and that grounded theories have potential to explain them in novel and insightful ways (Barsalou, 1999, 2008b, in press-b). As Binder (2016) suggests, another important function of association areas is to integrate conceptual information (also see Humphries, Binder, Medler, & Liebenthal, 2006; Legrand & Ruby, 2009).

Although these conceptual phenomena may seem dated, the only things dated are our theories of them. As current theories evolve, it’s essential that they continue to offer compelling accounts of foundational phenomena associated with conceptual processing.

## Making Contact with Real-World Situations

Cognitive psychologists typically assume that the kinds of phenomena listed in Goldinger et al. (2016) generalize readily to real-world situations. As a consequence, no attempt is made to assess these mechanisms in richer, more natural situations outside the idealized laboratory environments in which they were established. As Henrich, Heine, and Norenzayan (2010) found, however, such assumptions are not always warranted, given that a given cognitive mechanism can take different forms outside the laboratory.

The Visual World Paradigm offers another good example of how a basic cognitive phenomenon can change significantly when embedded in more realistic situations (Huettig et al., 2011; Tanenhaus et al., 1995). Prior to the visual world paradigm, what we knew about the processing of words and syntax was based on paradigms that largely examined them in isolation. Once researchers began studying them in the context of visual worlds, understanding evolved considerably to reflect substantial contributions from context.

Because concepts are typically studied in relatively idealized laboratory tasks, accounts of them may similarly be limited; we may be missing important phenomena and experiencing a variety of distortions. Although many of the basic conceptual phenomena established so far are likely to remain, it wouldn’t be surprising if new findings and insights followed as well.

As Rumiati and Foroni (2016) illustrate, food concepts offer an example of how concepts can be studied in a rich real-world domain (also see Papies, 2013; Papies & Barsalou, 2015; Ross & Murphy, 1999). Because eating is a multifaceted phenomenon that utilizes many cognitive processes, including attention, categorization, learning, decision making, goal pursuit, and affect, it offers the opportunity to examine how concepts operate in a rich cognitive context that also happens to be of social significance.

Many other real-world domains similarly offer useful contexts for increasing our understanding of how concepts operate in the real world. Barsalou et al. (2007) suggested two general types of situations that are central in human activity: (1) A single individual performs situated action in the physical environment to achieve a goal, as when building an object, navigating to a location, or gathering resources; (2) Multiple individuals perform coordinated social interaction, as when performing knowledge transfer, group decision making, or distributed operation of complex equipment. Studying how concepts support intelligent activity in situations like these might lead to new insights about their content, structure, and function, which wouldn’t emerge from studying them in the laboratory.

I’m not suggesting that basic cognitive research become applied. Instead, I’m suggesting that doing a better job of linking basic research to the real world is likely to have significant benefits for basic research. Bringing cognitive theory and results to bear on real-world problems will not only produce social benefits, it will increase the visibility of cognitive research and perhaps bring it greater appreciation and resources. Furthermore, examining basic cognitive processes in real world situations offers opportunities to assess whether our basic accounts are correct and complete, and if not, where further development is needed. To the extent that an account of a cognitive process is inadequate, assessing its behavior in the real world is likely to inform how the account could be revised, leading to important new basic research opportunities in the laboratory. In these manners, connecting basic research to important real world situations has significant potential to keep basic research from becoming insular, and to promote its productive growth and evolution.

## Conclusions

I began with Quixotic dead ends for studying concepts, namely, principled approaches that are unlikely to succeed. In this category, I included grounded theories that reduce to sensory-motor processes, grounded theories without representation or biological constraints, grounded theories that focus only on embodiment, and grounded theories that aim to replace standard accounts of cognition rather than complement them. In the category of Quixotic dead ends, I also included amodal theories with arbitrary symbols that can operate in a stand-alone manner, and approaches to cognitive psychology that simply develop lists of cognitive processes established in idealized laboratory paradigms unrelated to real-world situations.

Based on contributions to the special issue, I developed an approach to studying concepts that I believe has some chance of heading in the correct direction and enjoying some success. The properties of this approach include grounding, abstraction, flexibility, the ability to explain classic conceptual phenomena, and making contact with real-world situations. Having a few Quixotic bones in my body, I realize where I may be headed. Hopefully, I’m sufficiently grounded so that whatever my mistakes may be, they’re not too misleading and are useful for others in finding a clearer path.

## Footnotes

1 Although Mahon (2015) is not part of the special issue on Representation of Concepts, it is highly relevant and receives significant discussion.

2 An inherent contradiction exists in proposing that *amodal* processing areas reside in *modality-specific* processing streams. To the extent that the interaction hypothesis is correct, it makes no sense to call these streams “modality specific,” and new ways of referring to them would be required. When I discuss amodal areas in modality-specific streams here and at many later points, I’m simply referring to the possibility that amodal processing areas reside in what have *traditionally been called* modality-specific processing streams.

3 I do not include amodal systems for space, time, and magnitude in the class of amodal symbols that I’m concerned about. In spatial processing, for example, it’s possible that a single amodal system represents space across modalities (although this is far from agreed upon; e.g., Braga, Wilson, Sharp, Wise, & Leech, 2013; Farah, Wong, Monheit, & Morrow, 1989). Similarly, a single amodal system could represent magnitude across modalities (again this is far from agreed upon, contrary to Machery’s (2016) claim that consensus exists; e.g., Van Opstal & Verguts, 2013; Walsh, 2003; Yates, Loetscher, & Nicholls, 2012). It’s one thing, however, for amodal systems to represent information that is common across modalities, and another for amodal symbols to represent modality-specific information. It is only the latter that I do not believe reside in the brain. Later, in discussing distilled abstraction, I suggest that the brain represents many abstract features, similar to space and magnitude, that do not take the form of transduced amodal symbols (Barsalou, 1999).

4 Although Kemmerer (2015a) is part of the special issue on Representation of Concepts, it was published earlier in another issue of *Psychonomic Bulletin & Review*.
